# Type-IV Pilus Deformation Can Explain Retraction Behavior

**DOI:** 10.1371/journal.pone.0114613

**Published:** 2014-12-12

**Authors:** Ranajay Ghosh, Aloke Kumar, Ashkan Vaziri

**Affiliations:** 1 Department of Mechanical and Industrial Engineering, Northeastern University, Boston, Massachusetts, United States of America; 2 Department of Mechanical Engineering, University of Alberta, Edmonton, Alberta, Canada; University of Osnabrueck, Germany

## Abstract

Polymeric filament like type IV Pilus (TFP) can transfer forces in excess of 100 pN during their retraction before stalling, powering surface translocation(twitching). Single TFP level experiments have shown remarkable nonlinearity in the retraction behavior influenced by the external load as well as levels of PilT molecular motor protein. This includes reversal of motion near stall forces when the concentration of the PilT protein is loweblack significantly. In order to explain this behavior, we analyze the coupling of TFP elasticity and interfacial behavior with PilT kinetics. We model retraction as reaction controlled and elongation as transport controlled process. The reaction rates vary with TFP deformation which is modeled as a compound elastic body consisting of multiple helical strands under axial load. Elongation is controlled by monomer transport which suffer entrapment due to excess PilT in the cell periplasm. Our analysis shows excellent agreement with a host of experimental observations and we present a possible biophysical relevance of model parameters through a mechano-chemical stall force map.

## Introduction

**Figure 1:**
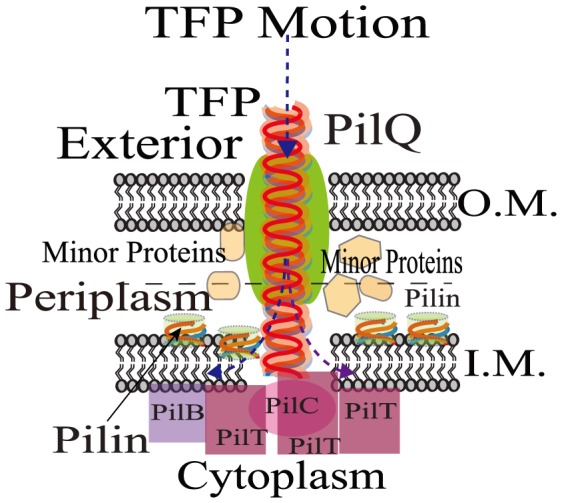
A schematic illustration of the protein ensemble near the TFP base inside the cell wall which are responsible for the retraction process. The retraction/elongation process involves a large number of minor and primary proteins such as PilT, PilB molecular motors, PilC platform protein as well as the pore PilQ, all spread across the periplasm of the cell. Pilins are stowed in the inner membrane after de-polymerization(retraction)and are subsequently recruited during polymerization (elongation). Dashed arrows indicate direction of motion. O.M.: Outer Membrane and I.M.: Inner Membrane [9], [18], [28].

Elongation, adhesion and retraction of long polymeric nano-fiber called type-IV pilus (TFP) results in a form of bacterial surface translocation called twitching motility which causes complex colonization events such as virulence, biofilm formation and fruiting bodies [1]–[3]. A host of proteins including molecular motors aid twitching motility through mechano-chemical processing of TFP, Fig. 1 [1], [2], [4]–[8]. This highly repetitive processing consisting of rapid de-polymerization of TFP into pilins and the reverse - polymerization of the pilins into TFP near its base has been directly observed in *Pseudomonas aerginosa* [9]. Among the ensemble of proteins responsible for TFP processing, the crucial role PilT protein [10], [11], a molecular motor, in aiding retraction was unambiguously isolated and quantified in *Neisseria gonorrhoeae* [3]. The *in vivo* TFP retraction force-velocity characteristic of *N. gonorrhoeae* loaded using laser trapped micro bead showed constant retraction velocity at lower forces which then decayed to a stable indefinite stall as load was increased [12]. Interestingly, the retraction force-velocity characteristic was found to be nearly identical for mutants with differing concentration of PilT or periplasmic pilin. Later experiments on *N. gonorrhoeae* using similar set up showed that TFP retraction may even be reversed at stall fairly quickly into elongation for mutants with low concentration of PilT [13]. More recent studies on *N. gonorrhoeae* have shown an yet undiscovered higher retraction velocity at lower forces for high PilT concentration mutants [14]. Thus, although the overall role of PilT protein in fostering TFP processing is beyond scrutiny, the exact interplay between force and PilT in altering force-retraction/elongation characteristic is intriguing thereby requiring assumptions beyond simple Arrhenius type kinetics [14], dynamics of a single Brownian motor or polymer ratchet mechanisms [15]. In this paper, we show that in contrast to the direct effect of force, the elasticity and geometry of the TFP together with its interfacial behavior when coupled with chemical kinetics play a key role in explaining the experimentally observed characteristics. This mechano-chemical paradigm which shows that retraction behavior is influenced by the characteristic of both the molecular motor and the TFP therefore point towards their *coevolution* whose strong evidence for *N. gonorrhoeae* has been reported in recent experiments [16].

## Analysis

**Figure 2:**
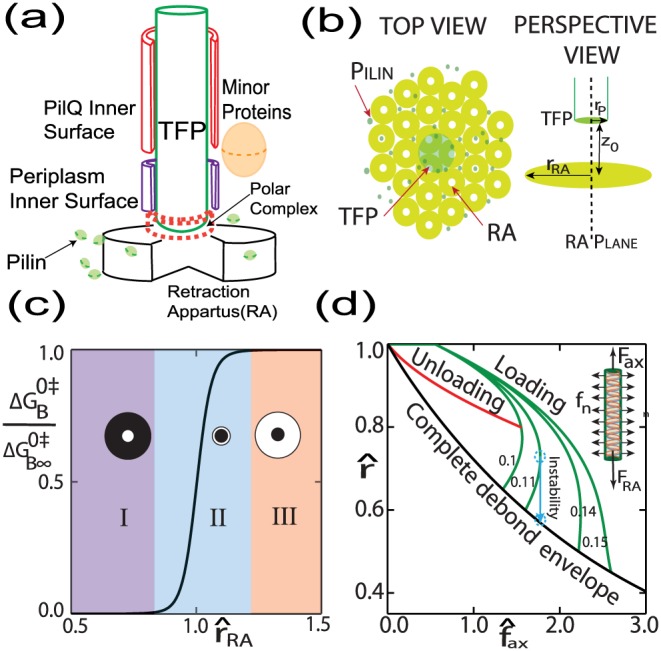
Model simplification of TFP biological apparatus and their consequences. (a) A simplified reduction of the TFP processing bio-system into an axi-symmetric structure with a sliced view of TFP-protein/periplasm interfaces. The cylindrical retraction apparatus(RA) sits below the TFP base on the cytoplasmic part of the cell and the shallow cylindrical polar complex at the end of the TFP(shown in dotted red lines) is an electrostatic complex which is essential for recruiting pilins for elongation [18], [31] (b) the top part of the RA (only PilT shown) forming the RA-plane is responsible for the binding regime of the retraction process and is assumed to be very closely packed with PilT units sitting close to the base of the TFP. Note that the empty space surrounding the TFP and above the RA plane in this figure is actually filled by PilQ, enclosing periplasm and embedded minor proteins. (c) binding energy at zero deformation as a function of size of the RA-plane indicating three distinct zones and a strongly saturating characteristic assuming a van-derWalls type binding. The x axis is RA radius normalized by the pilus radius and y-axis is current binding energy normalized by that of an infinite plane. (Inserts: White circle indicates the size of RA plane and black the TFP cross section). (d)Normalized force-radius characteristic of TFP. The numbers on the loading curve (green) represent 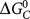 (Insert: Free body diagram of loaded TFP, 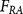 is binding force due to RA).

We first simplify the cell wall portion of TFP bio-system illustrated in Fig. 1(b) into an equivalent homogenized axially loaded axi-symmetric cylindrical structure, Fig. 2 (a). The TFP is surrounded by a large protein PilQ spanning about half of periplasm, minor proteins as well as the periplasmic material itself [17]. These minor proteins include for instance in *N. gonorrhoeae*, PilD which is a preplin peptidase [18] without which the bacterial will not be able to process the incipient pre-pilin into pilin subunits [19], PilG which is another crucial inner membrane protein closely related to PilD and also aids in pilus biogenesis [20], PilF which is an assembly ATPase without which the bacteria would not be able to assemble the mature pilin subunit [19] and PilC which acts as a tip-located adhesin for end attachment of TFP useful for instance in DNA uptake [21], [22]. The morphology of PilQ protein found widely in various gram negative species [18] is most well characterized in *Neisseria meningitidis* [18] where a four-fold symmetric cage like structure emerges through cryo-electron microscopy (EM) reconstruction [23]. A side view resembles a cylindrical hollow frustum with a tapering cavity which narrows down somewhat towards the bottom [23]. Absence of this elaborate pore would leave no place for the assembled TFP to emanate from the cell [24]. Interestingly, the binding capabilities of this protein for long helical DNA strands for both *N. meningitidis* and *N. gonorrhoeae* have been well known [25], [26] and the similarity of the machinery with TFP processing has been already theorized [3]. This suggests that the inner surface TFP-PilQ interaction is dominated by a radial adhesive traction field.

The TFP base may host a *polar complex*(PC) which propels pilin recruitment through the charged end of growing TFP during elongation [18]. The exact nature of the retraction process is still under intense scrutiny although molecular motor PilT is well known to play a crucial final part in force generation as discussed earlier. It has been speculated that either single [12] or several units may be acting in unison [15]. It is also believed that several minor proteins do play important role in the dis-assembly forming a biological complex near the TFP base [13], [27]. We simplify the arrangement of retraction proteins into a self-assembled axi-symmetric ensemble called retraction apparatus (RA) where motor proteins such as PilT play a leading role together with ancillary proteins such as PilC in TFP dis-assembly [18], [28]. PilT is a hollow cylinder which binds with the TFP at one end, excreting pilins at the other through large domain motion utilizing ATP hydrolysis [10], [11]. This TFP consumption kinetics can be idealized as taking place in two steps via two distinct transition states (TS)-the first TS is part of the binding step which results in a metastable intermediate structure bound to the RA. The activation free energy for this reaction is mostly enthalpic in nature due to the binding field. This meta-stable structure then disintegrates into pilins via another TS with the aid of PilT to mark the processing step which is likely entropic in nature due to polymer dismemberment and is independent of the binding field. The binding step determines the rate of forward and the processing step determines the rate of backward reaction. Note that the kinetics subsumes the exact details of the still unclear molecular mechanism of this transformation process involving a plethora of long and short range forces, interacting chemical species as well as thermally induced motion in highly complex condensed media through a unified reaction coordinate. We idealize the binding as taking place between a sheet of binder surface and the end cross section of the TFP with uniformly distributed binder sites, Fig. 2(b). In order to compute the binding free energy 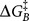 (calculated per molecule of TFP material) we assume a vanderWalls(vdW) type interaction [29] which is represented by an inverse sixth power pair potential when the surfaces are sufficiently away from the steric repulsion regime (see Fig. 2(c)):
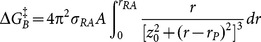
(1)here 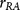 is the radius of the retraction apparatus plane, 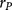 is the current radius of the TFP, 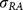 is areal density the binding site on the RA surface, 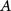 is the vdW binding constant and 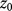 is the inter-surface binding distance taken roughly equal to an average pilin characteristic length of 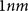 [30]. Eq. 1 can be re-written in terms of lengths normalized by TFP radius 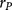 and plotted for various values of normalized RA radius 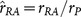, with 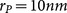 [30], Fig. 2(c). This plot exhibits a strong saturation characteristic, i.e. 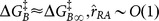 where 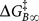 is the binding free energy of an infinite plane i.e. 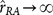. Since the diameter of PilT is roughly of the order of the TFP itself [10], [18], from the geometry of the set up we conclude that increasing the concentration of PilT which would amount to increasing the size of RA will have little long term effect on retraction behavior as repeatedly confirmed in experiments [12], [13]. Furthermore, evaluation of Eq. 1 in the infinite plane limit would yield:
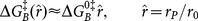
(2)where 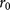 is the undeformed TFP radius and the superscript 0 indicates the binding free energy under standard conditions of zero deformation, i.e. 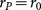. Note that 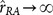 would lead to an apparent geometric incompatibility since the bacterial surface is curved. However, due to relatively large radius of curvature of the nearly spherical bacterial surface in comparison to TFP cross sectional dimensions and the rapidly attenuating nature of the pair potential, the effect of curvature is deemed negligible in our analysis. Thus the net areal mass production rate at TFP base for the retraction process 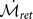 assuming unit chemical activity for TFP and pilin material would be:
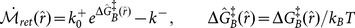
(3)where 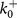 is the rate constant without binding for the forward process, 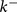 is the rate constant for the backward process, 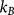 is the Boltzmann constant and 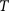 is the temperature. Note the TFP retraction velocity 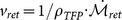 where 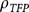 is TFP mass density. In contrast to retraction process, elongation involves both polymerization and pilin transport towards the base of the TFP propelled by the electrostatic forces at the PC [18], Fig. 2(a). The PC however, must itself be stabilized for a steady pilin transport [31]. We propose that the stabilization is possible only when the net retraction rate has been diminished sufficiently. Once the incipient nucleus of the PC has stabilized, mass transport towards the TFP base commences resulting in the following flux- controlled elongation areal mass transport rate 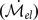:
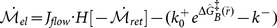
(4)where 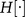 is the discrete Heaviside step function and 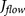 is out of RA plane transport flux at TFP base assumed approximately constant in the current study. Clearly, this thermodynamic framework depends on the TFP radius. In order to determine the evolution of TFP radius, we employ an elastic analysis of TFP deformation. To this end, first note that TFP is a multi-stranded helical structure [18], [30]. Although some axial variation in geometry is possible, we approximate it as a regular 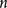-start helical structure with a constant helix angle. A typical TFP with an outer radius of 10 nm and inner radius of about 5 nm [30]under about 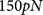 of peak stall force [13] would be under a mean axial stress of less than 1 MPa at a near stall loading rate of less than 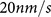 [13] implying negligible inelastic effects. Furthermore, electrostatic and thermal contribution to the strain energy are also neglected. In addition to the axial loading force, there are radial adhesive forces on the structure due to the volume surrounding the TFP as it runs through the enclosing PilQ, minor proteins as well as periplasmic gel, Fig. 2(a) [17]. For simplicity, an average uniform adhesive traction is taken. All interfaces are assumed frictionless.

**Figure 3:**
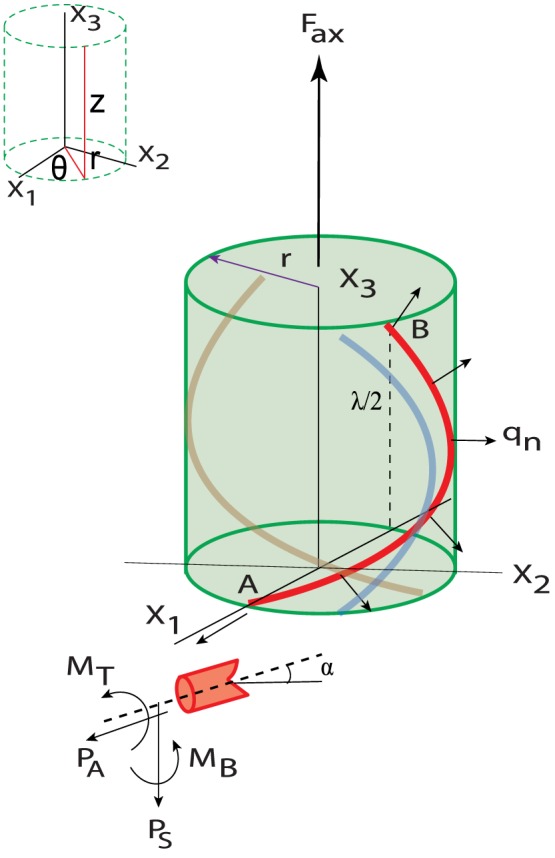
Free body diagram of the section of the homogenized cylindrical type 4 pilus (TFP) with individual strands shown as colored filaments. A section of an individual filament is depicted in the lower left corner. (Insert: Cylindrical coordinates).

A free body diagram of the pilus depicting all the forces is now shown Fig. 3. Without loss of generality, a Cartesian coordinate system has been attached to the system depicted by the triplet 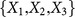. A slice of a filament at helix half-rise 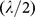 is taken to show the internal forces and moments acting on one of the filaments. The external applied axial force is shown as 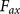. The internal forces are respectively the axial 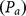 and shear forces 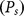 as well as bending moment 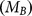 and twisting torque 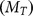. In addition, the uniform cohesive traction 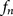 is assumed to act on the homogenized cylinder. The total force per unit pitch (pitch length 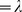) is thus given by 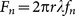 where 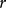 is the radius of the helix. This same force can be written in the form of a force per unit length of an individual filament 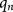 as 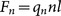 where n is the number of strands (starts) in the helical structure and 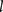 is the length of these strands enclosed within a pitch 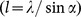. Thus we get a relationship between the traction and the force per unit length as:
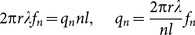
(5)


Neglecting inertia and using the balance of moments about A in the 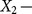
direction (see Fig. 3) we get:
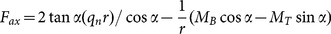
(6)


Assuming linear elasticity and inextensibility (small strain) for the filaments together with Euler-Bernoulli kinematics, the moment equations can be written as:
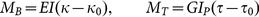
(7)


Where 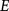 and 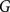 are the Youngs and Shear modulus of the filament, 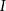 and 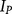 are the transverse and polar area moment of inertia of the filament cross section, 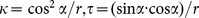 are the principal curvature and twist of the helix respectively and zero subscripts denote the values in the initial configuration. Furthermore, assuming no unwinding takes place, we have, 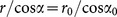 where 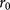 and 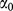 are initial radius and pitch angle respectively. Using no unwinding together with Eq. 5 and Eq. 7 in Eq. 6 along with the expressions for 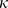 and 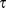, we get the following normalized force expression:
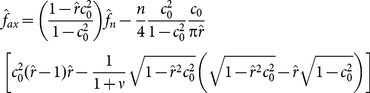
(8)where 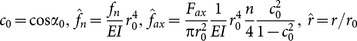 and 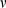 is the Poisson's ratio. The above can be further re-written as:
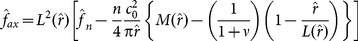
(9)


The normalized adhesive traction 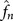 which is a result of the combination of contact forces exerted by the inner surface of periplasmic gel with the TFP surface and the radial adhesive traction due to TFP-PilQ inner surface as well as the long range forces exerted by minor proteins discussed earlier is modeled using the following axially uniform traction-separation law [32]:
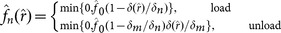
(10)where 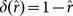, 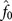 is the normalized adhesive strength of the interface, 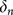 is a dimensionless separation at complete failure and 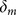 is the dimensionless separation at maximum load in case of partial failure. This relationship implicitly implies that the separation at which cohesive strength is reached, 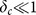 and thus non-dimensional interface cohesive free energy 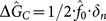.

## Results and Discussions

**Figure 4:**
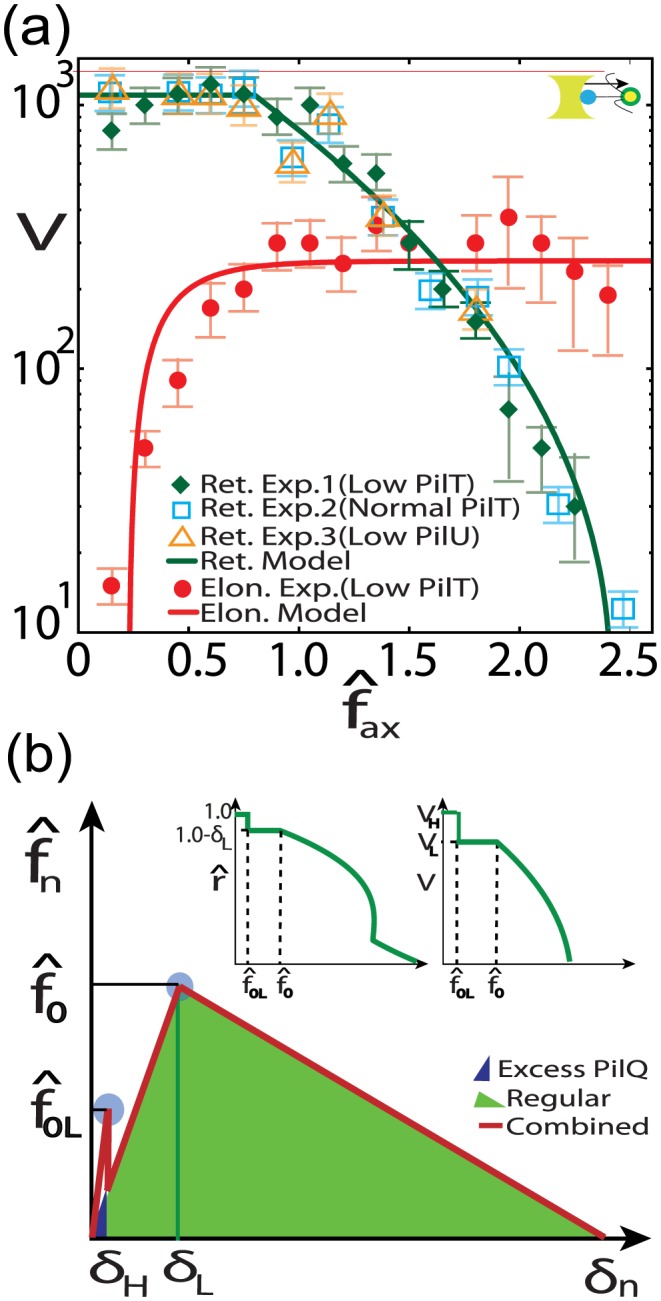
Comparison with experiments. (a)Force-Bead velocity (in 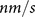)comparison of the model with experiments [13](Top Insert: Experimental setup)(b)traction-separation diagram showing progressive debonding 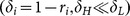 and its effect on force-radius and force-velocity relationship which is now capable of reproducing the higher velocity mode of retraction 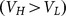. 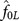 is the lower cohesive strength(Inserts: Effect of progressive debonding on force-radius and force-velocity characteristics).

Taking the geometrical properties of a typical *N. gonorrhoeae* TFP, we have 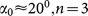 [30]. In addition, assuming a Poisson's ratio of 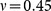 and 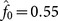, we generate the force-radius characteristic parameterized by 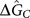, Fig. 2(d). From here it is clear that lower 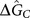 can result in material instabilities providing an instantaneous path for switchover from one branch to another thereby speeding the retraction-elongation switch as observed experimentally [13]. The portion of TFP external to the bacteria which is already under hydrostatic external pressure of the medium has been assumed pre-stretched by the time of debonding and thus does not contribute significantly to the retraction velocity. Although exact elastic parameters needed in the model have not been reported, we make indirect deductions. For instance, extension experiments on single TFP [16] have shown roughly a 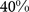 diametric reduction at forces of about 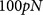. Thus from Fig. 3(a), 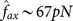. With these values, and using the following set of fitting parameters: 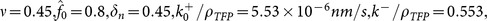 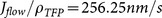 and 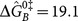 in Eq. (3–5), we compare our model with single pilus elongation-retraction experiments [13] in Fig. 4(a) (TFP geometrical properties have been kept as before) and find excellent agreement. Furthermore, in agreement with experiments [13], retraction would resume as soon as laser trap is switched off since deformation vanishes causing instantaneous increase in radius and thus de-polymerization rate (Eq. 3). Also, it has been found that only bacterial strains with low PilT concentration exhibit elongation but with indistinguishable retraction behavior when compared with normal or high PilT concentration strains [13].

This is a characteristic of our model where the elongation can be significantly attenuated by increasing levels of PilT in the inner membrane due to increased pilin entrapment by PilT during transport. In the case where elongation is no longer possible due to a precipitous drop in pilin transport, the stall would represent a stable equilibrium. Although, purely concentration based diffusive transport has been ruled out since retraction rate was found to be indifferent to either the length of the retracted TFP or levels of pilin [12], any general transport process which suffers pilin entrapment due to PilT distribution in the periplasm would still exhibit this attenuation phenomena. The simplest model of uniform entrapment sites will lead to an exponential drop in mass transport rate with transporting distance [33] and thus, a higher level of PilT would also lead to much greater pilin entrapment leading to an eventual extinction of the incoming pilin mass flux beyond a threshold PilT concentration. Interestingly, areal density of entrapment sites would be directly related to only PilT units since they have a natural binding affinity for pilins and therefore, other co-expressed proteins (such as PilU) will have little effect on elongation; a claim which has already been confirmed by careful experiments [13].

Interestingly this transport step which involves material transportation is slower than reaction and thus elongation process will exhibit pauses to allow for pilin buildup at TFP base, another observed hallmark [13]. Recently, a higher far-from-stall retraction velocity (almost twice the average reported earlier)was observed at lower forces and high PilT concentration which abruptly switched to the widely observed lower retraction velocity as loading was increased [14]. We propose that excessive concentration of PilT causes an additional ring of PilTs to build up above the RA plane surrounding the TFP which although does not contribute to the retraction kinetics due to adverse steric position does provide another cohesive energy profile to the TFP. This profile is typically much weaker and more brittle than the existing profile due to poor contact and therefore alters the traction-separation law into a progressive one as shown in Fig. 4(b). Therefore, instead of a single separation at which cohesive strength is reached i.e. 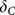, there are two such separations: 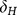 corresponding to the weaker PilT interface and 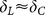 correpsonding to the usual interface. Thus at 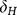 TFP radius is 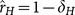 resulting in binding energy 
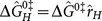
. Similarly, at 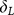, the binding energy is 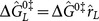. From Eq. 2 and Eq. 3, we get 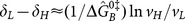 where 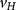 and 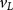 are respectively the retraction velocities (far from stall) of the higher and lower modes. Holding other model parameters constant, assuming 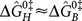 and using experimental values [13], [14] we get 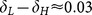, implying 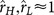, and thus 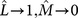 in Eq. 9. Therefore, this modification simply adds another step to the force-retraction curve at lower forces, Fig. 3(b), thereby explaining the bimodal switching behavior. Note that due to inherently weak nature of this additional interface, this mode would be difficult to observe or sustain thus escaping detection in earlier ‘spring loaded’ experiments [14].

**Figure 5:**
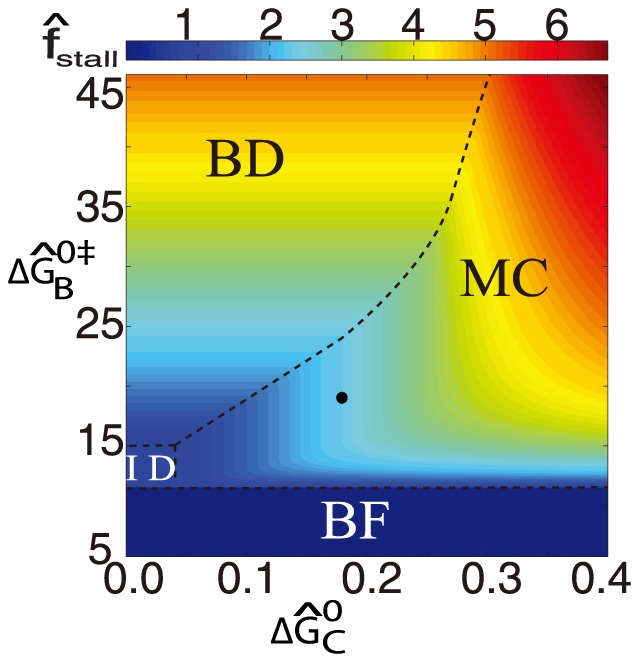
Stall-map indicating variation of normalized stall force with mechanical cohesive energy and normalized chemical binding activation free energy. Dashed lines show phase boundaries. The black circle shows the location corresponding to the experiments [12], [13]. BF: Binding Failure, MC: Mechano-Chemical, BD: Binding Dominated and ID: Interface Dominated.

We now generate a mechano-chemical stall plot in Fig. 5 which shows the landscape of normalized stall force variation depending upon 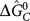 and 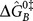 while other parameters are held constant from above. In this phase plot, at the bottom lies a binding failure region characterized by very low binding energy where retraction is decimated. As binding improves, we come across the next transitory interface dominated regime where binding energy is only large enough to be offset rapidly as soon as the interface fails, thereby constraining stall force to be near interface strength. As binding energy increases further, a binding dominated region emerges, where the stall force monotonically improves irrespective to the characteristic of the TFP interface. Bordering these regions lies the *mechano-chemical* region where there is a complex interplay of the cohesive and the binding energy making it possible to arrive at a stall force through a relatively small variation of properties of both TFP interface and molecular motor. Since higher levels of PilT can produce additional weaker interfaces as well, this region provides maximum gains through PilT concentration changes. More specifically, in this region, poor alignment of PilT units due to excessive crowding which can otherwise reduce binding free energy and thus stall force may be mitigated automatically through additional cohesive energy. Thus the stall force which is an important parameter for survival and replication of these bacteria including biofilm formation and virulence [3] is much more robust in this mechano-chemical region. It is in this region that the experiments conducted on *N. gonorrhoeae* [12], [13] lie and we believe this to be no coincidence as it boosts the evolutionatry adaptability of the organism. Furthermore, this region also provides a strong biophysical basis for *coevolution* of both TFP properties as the underlying molecular motors, reported recently [16].

## Conclusions

To conclude we have developed a simplified but biophysically consistent model to understand the behavior of the TFP retraction behavior which includes the pilus deformation. We discover that inclusion of TFP deformation along with an interplay between its surface-interfacial and end-binding behavior plays a key role in explaining a host of yet unexplained experimental behaviors. This includes the excellent quantitative reproduction of the experimentally observed force-velocity curves, force induced switching of retraction to elongation only at depressed levels of PilT, the instantaneous reversion to retraction when optical trap is turned-off, the apparent asymmetry between retraction and elongation in the velocity profile, the relative independence of retraction and elongation behavior on PilU or PilE (pilin) levels and a possible reason for an elusive bi-modal retraction velocity profile. Furthermore, this deformation based model which is used to construct an energy phase diagram mapping the experimental locus on a interfacial-binding energy axis. This phase map was shown to provide a possible explanation for the observed co-evolution between the molecular motors and the TFP itself. Note that although the experiments yielding the parameters were conducted on *N. gonorrhoeae*, TFP processing system is known to be extremely primitive and thus shows similar properties across a wide gamut of bacterial species thriving in widely different environmental landscape [14]. Hence, conclusions drawn here are of broader biological significance.
